# Desensitization in transplantation: is intravenous immunoglobulin the holy grail?

**DOI:** 10.1590/2175-8239-JBN-2022-E010en

**Published:** 2022-12-09

**Authors:** Ragnar Palsson, Leonardo V. Riella

**Affiliations:** 1 Massachusetts General Hospital, Department of Medicine, Nephrology Division, Boston, MA, USA; 2 Harvard Medical School, Boston, MA, USA

Patients with kidney failure who undergo kidney transplantation have better survival and quality of life than those managed with dialysis.^1^,^2^ Sensitization to human leukocyte antigens (HLA), resulting from pregnancies, exposure to blood products, or prior transplants, can become a significant obstacle to transplantation for patients with kidney failure. Highly sensitized patients are likely to have difficulty finding a suitable kidney donor against whom they do not have one or more anti-HLA donor-specific antibodies (DSA). Kidney transplantation in the presence of DSA, particularly with a positive physical crossmatch, carries a high risk of early allograft loss from antibody-mediated rejection (ABMR). Furthermore, even when early ABMR may be avoided, patients receiving transplants under these circumstances are at higher risk of chronic ABMR. Together, these complications lead to shorter allograft survival among kidney transplant recipients with DSA compared to that of recipients without DSA.^3^ To maximize graft survival, kidney transplantation across a DSA barrier is thus avoided whenever possible. As a result, highly sensitized patients are on the waiting list for kidney transplant longer and achieve lower rates of transplantation than non-sensitized patients.

Various efforts have been made to improve access to kidney transplantation of highly sensitized patients. In the United States, sensitized patients are given priority in the national kidney allocation system when well-matched organs become available. A major expansion of kidney donor exchange programs has also helped sensitized patients find suitable living donors. An alternative or sometimes additional method to overcome the transplantation barrier of highly sensitized patients is to attempt desensitization.^4^ Desensitization in transplantation is actually a misnomer, since we cannot make patients nonreactive or insensitive to antigens against which they have previously developed anti-HLA antibodies. The goal of desensitization therapy is to lower the immunological risk of the potential kidney transplant recipient sufficiently to avoid ABMR and early graft loss by reducing anti-HLA antibody levels. Since the emergence of desensitization for HLA incompatibility in the 1990s, various methods serving this purpose have been described. Intravenous immunoglobulin (IVIG) has remained a cornerstone of de-sensitization protocols since their inception and is typically administered either via high-dose infusions or, when accompanying plasmapheresis, through more frequent lower-dose infusions.

IVIG has widespread immunomodulatory effects, affecting most immune cells and influencing levels of antibodies, complement activation, and cytokines.^5^ After being discovered to have therapeutic effect in several autoimmune disorders, its potential role as a desensitization agent was subsequently identified^6^. In an early study of IVIG given as monotherapy for desensitization, 15 highly sensitized patients received three monthly IVIG infusions at a dose of 2 g/kg, of whom 13 were effectively desensitized and able to subsequently undergo transplant.

One allograft was lost because of acute rejection and 1 due to thrombosis, but the remaining 11 recipients had good 1-year outcomes without rejection. The efficacy of IVIG as a desensitization agent was then more definitively demonstrated in a multicenter randomized placebo-controlled trial in which highly sensitized patients were given monthly infusions of either IVIG (2 g/kg) or placebo, and rates of transplantation were compared. Those who received the high-dose IVIG infusions had higher rates of both living and deceased donor kidney transplantation than those who received placebo (35% vs. 17% and 31% vs. 12%, respectively, p<0.05 for both comparisons). However, graft survival among transplanted patients did not differ between those who received IVIG and those who received placebo^7^. In the study published in the *Brazilian Journal of Nephrology* by Ulisses *et al.* the authors re-visited the use of IVIG monotherapy for desensitization. By administering monthly 2-g/kg infusions of IVIG, 29 of 33 highly sensitized patients with median panel reactive antibodies (PRA) >80% were able to undergo transplantation after a median of 6 months. Death-censored graft survival was 79.2% at 5 years, with allograft loss being attributed to chronic ABMR in 40% of cases.^8^

But does the existing literature support IVIG monotherapy as an optimal desensitization method? We are of the opinion that it does not. Most studies describing desensitization protocols have significant intrinsic limitations and cannot be directly compared due to their heterogeneity. These studies are usually descriptions of single-center experiences that include small samples of patients, whose HLA testing and immunologic risk assessment is carried out in a non-standardized and variably precise manner. Importantly, appropriate control groups and data on long-term patient outcomes are often lacking. In one of the rare studies directly comparing different methods of desensitization, Stegall *et al*. showed that patients that received high-dose IVIG alone, albeit in a single dose rather than repetitively, had much lower chances of achieving a negative crossmatch and higher risk of rejection post-transplant than patients whose desensitization consisted of low-dose IVIG, plasmapheresis, and rituximab.^9^ Vo *et al*. aimed to rigorously evaluate the efficacy of adding rituximab to high-dose IVIG for desensitization in a randomized placebo-controlled trial. Their study, initially designed to include 90 patients, unfortunately had to be stopped early, as 5 serious adverse events were observed among the first 13 transplanted patients, 7 of whom had been randomized to the placebo arm. Two of these events involved graft loss and 3 were ABMR episodes. When the study was unblinded, it was found that all of the events occurred among the group receiving high-dose IVIG alone. While statistical power was limited, patients who received the combination of IVIG and rituximab also had significantly better allograft function at 6 and 12 months than those who received IVIG alone.^10^

**Figure 1:**
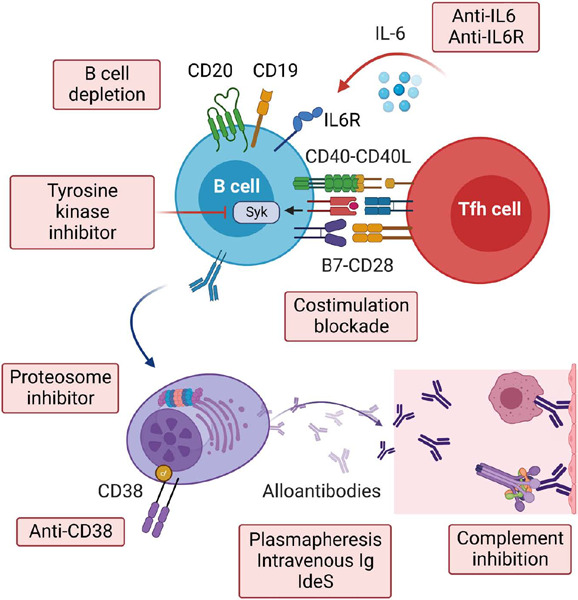
Drugs targeting multiple steps involved in anti-HLA antibody generation, maintenance, and effector function, including B cell activation, plasma cell survival, circulating antibodies, and antibody-mediated endothelial injury.

While past studies such as these suggest that highly sensitized patients benefit from a multifaceted desensitization approach, all advances to improve access of this group to kidney transplantation are welcome. Plasmapheresis and adjunctive medications such as rituximab are costly and not universally available. Simplified desensitization protocols, if sufficiently effective, may still offer highly sensitized patients net clinical benefit by minimizing their exposure to dialysis. With multiple new approaches for desensitization and treatment of ABMR on the horizon, such as alternative anti-CD20 monoclonal antibodies, interleukin-6 blockade, anti-CD38 monoclonal antibodies, and the cysteine protease imlifidase that cleaves pre-formed IgG (Figure 1), our ability to address the disadvantage of highly sensitized patients may soon improve. Major persisting challenges with desensitization are the post-transplant high antibody-mediated rejection rate (20-60%), lack of efficacy in very highly sensitized recipients (PRA>98%), high cost, and worse long-term graft survival. For kidney transplant candidates with incompatible donors due to DSA, kidney paired exchange should be the primary choice. For patients without a living donor, desensitization permits expanding the potential pool of compatible deceased donors. For them, the key to progress lies in conducting long overdue, well-designed, and adequately powered randomized controlled trials with a multi-target approach that aims to both reduce circulating anti-HLA antibody levels and inhibit further generation of antibodies by B cells and plasma cells.
